# Pyorrhea*Read before the Tennessee State Dental Association, June 5, 1913.

**Published:** 1913-08-15

**Authors:** Claude E. Hines

**Affiliations:** Memphis, Tenn.


### PYORRHEA.*

BY CLAUDE E. HINES, D.D.S., MEMPHIS, TENN.

Whenever asked to give the cause of pyorrhea, I have to reply, "I do not know the primary cause," and then by way of an explanation for my ignorance follow with the remark that "I don't believe anyone else does." The secondary or exciting cause of pyorrhea is the deposit of tartar salivary or serumal. To this may be added, the failure of the dentist to properly clean the teeth, ill-fitting crowns and bridges, fillings which have no contact point, or which have overhanging margines, and failure to observe and correct mal-occlusions.

There are many things, the why and the wherefore, of which our finite minds are not able to solve, yet we know that they exist. Pyorrhea is one of them. Like the great white plague, we see its ravages daily. Therefore, it is our duty to warn of its danger, and battle with it to the last ditch. Because we don't know the exact cause of a thing is no reason why we should not take cognizance of it. We never knew the cause of caries till long after we knew how to fill teeth.

I have no patience with the theory that a man must "specialize" in order to combat this disease. There are thousands of people who need our services in this direction who can never see inside of a specialist's office. Are we doing our best as a profession when we are careless in our views on so important a subject?

Do you know most of us have taken the bull by the tail instead of the horns; we have learned how to construct beautiful bridges, fillings and inlays; we are trying to learn how to put in silicate fillings, and we build all this beautiful work over what?—over a mass of muck, reeking with every germ that has so far been catalogued. Get in front of the

*Read before the Tennessee State Dental Association, June 5, 1913.

bull! Stop telling the public that a one dollar cleaning is all they need! Stop saying, you will charge them for their work and "throw in" the cleaning! Let me tell you the one dollar and "throw in" cleanings have been the cause of more edentulous mouths than anything I know of. I don't care how well a tooth may be filled, how good a crown you have placed on it, or whatever your work may have been, if you have failed to remove any deposit on the root of that tooth, your work is in constant danger.

The public is becoming educated. They are beginning to realize that three-fourths of the trouble in their mouths is caused by our neglect. They are beginning to know that the one dollar and "throw in" cleanings are a delusion and a snare; they may accept them, but they realize more and more that they are not getting the worth of the time spent much less their money's worth.

### TREATMENT.

I believe in preventive measures. I believe in prophylaxis. I believe that the time to recognize and treat pyorrhea is when we CLEAN a patient's teeth. I believe that the time to clean teeth is when we first see the patient, unless that patient be in actual pain from some other cause. I believe that "cleanliness is next to Godliness," and this applies not only in the handling of this disease from its first to last stages, but must be put into practice by the patient ever afterwards. Co-operation between patient and dentist means success! If you can't make them understand that point the sooner you dismiss them the better.

Pyorrhea is curable just as tuberculosis is curable, and the fact that disaster overtakes some cases is no more of an argument against its cure than the fact that death overtakes many a case of tuberculosis. Physicians endeavor to cure—if they fail, they try to give relief as long as possible, and it is our business to do likewise.

Away with the idea that pyorrhea is incurable! Get to work and watch the transformation from disease to health in the mouth of some patient, and you will agree with me, it's about the best looking sight you've ever seen.

The treatment of pyorrhea may, for convenience sake, be divided into three divisions: surgical, medicinal and mechanical. To my mind the three are inseparable. To get the best results in all cases we must be thoroughly familiar with each of these procedures.

I do not agree with the idea that you must have a "special gift" or "special touch" in order to treat this disease, but rather think that your common sense and good judgment, coupled with a little hard work will be the only requirement necessary.

The surgeon when he operates prepares the patient both mentally and physically, if it is possible. I see no reason why the dentist should not use the same good judgment and sense when he goes to scale or clean the teeth of his patient. He is to work in a field filled with every kind of bacteria, known and unknown; he is to clean out accumulations of, possibly, years; he is to dig into places compared with which a sewer is a palace—then why not disinfect the field to begin with—why not sterilize the mouth as nearly as possible before you begin your work? The surgeon does; it would be well for us to follow his example.

Instrumentation for the removal of deposits should be positive and thorough—by this I mean, scale one tooth at a time and stick to it until you are sure it is clean.

Instruments for our use have been devised by the hundreds; some good, some bad—many of them positively worthless. The simplest form that can be used is the best, and for that reason I think most of the work should be done with files. Don't understand me to say that scalers are not useful; they are, but the use of many of them is positively detrimental to the result for which we are striving. Especially is this true of the long cow-horn pattern, made to cut on both sides. In using them we unnecessarily wound the tissues, and thus increase the area of infection. The only use we have for scalers is in the removal of salivary deposits, and they should be of the right angle, pull cut pattern. As a rule I do not believe in using any form of scalers in the pockets, but of course conditions arise where this may be necessary. Let me say here that I regard it as an imperative and unalterable rule to always follow the use of scalers with files. I believe that many failures in the treatment of this disease is caused by the failure to observe this one point. Scalers invariably leave a roughened surface wherever used, whether you can see it or not, and the only way to remedy this is to smooth the surface of the roots with files. Gum tissue will not adhere to a roughened surface; for this reason it is important that we leave the surfaces operated on as smooth as it is possible to make them.

### INSTRUMENTS.

A set of instruments adapted to our needs must possess many qualities. They must be few in number, capable of reaching every root surface, made of the best material, with handles of aliminum to transmit the sense of touch, and withall, capable of doing the delicate work for which they were designed. The Towner files come nearer meeting this requirement than any so far manufactured. The set consists of twelve instruments, four for the incisors, four for the bicuspids and four for the molars. In using these instruments it is well to bear in mind that each is made for a particular root surface, and to use them at that point contributes to our own ease in their handling. Of course, there are exceptions to this rule; for instance, working by mirror on the lingual surfaces of the upper incisors, the files made for the bicuspids are very useful. On the lower incisors, where the teeth are long, some of the files used in the molar region will be found of value. But the nearer we come to using them on the surfaces for which they are intended the greater will be our skill.

Our instrumentation should be thorough. We should take one tooth at a time and thoroughly clean the surface of the root. If there is no disease present, the instrument will barely enter under the free margin of the gum; if there is a diseased condition your instrument will tell you, for it will drop right into the pocket.

By way of parenthesis let me say that many mouths have diseased conditions around the roots of the teeth that show no outward evidence of it whatever, and the certain way to find it is by careful instrumentation. I have seen pericemental abscesses on teeth where the margin of the gum looked to be perfectly healthy.

Files have another value that should not be overlooked. While cleaning the surface of the root the stroke of the instrument polishes the crown of the tooth, so that when we come to polish with the brush and pumice the time for that work is materially lessened.

### MEDICINAL TREATMENT.

No medicine or formula of drugs has ever cured pyorrhea. Let me make that plain—dependance on drugs alone is folly. But drugs as an aid to a cure are indispensable.

Like our instrumentation our medication should be positive and simple, and then let nature do its work. Stop for a moment and let us consider the condition that presents itself when we have completed our scaling. We have opened up a hot-bed, a nest, so to speak of germs. True, we have removed many of them, but many remain. Race suicide is a thing unknown in germ life, so common sense would teach us to use something to kill the germ and stop the breed. We should use a germicide that is powerful yet harmless to the soft tissues.

Now, what germicide is best? That is the question for us to consider. Materia Medica gives us many powerful germicides, but their use is contraindicated on account of their destructive effect on the soft tissues. Some of these drugs are strong acids; their use causes not only a destruction of the soft tissues, but makes the teeth become extremely sensitive, causing the patient unnecessary pain and discomfort. I don't believe many conditions arise where the use of such an acid is necessary.

R-10.

In the first part of this paper I made mention of the fact that I believed we should sterilize the mouth as nearly as possible before we commenced our scaling. This I do by making an application of R-10 around the teeth to be operated on. In cases of acute inflammation of the gums I sometimes apply this a day or two before I operate to reduce the inflammation as much as possible.

The formula was devised by myself to meet a demand for a powerful germicide yet one that would have a kindly effect on the tissues. Laboratory tests have demonstrated its germicidal powers conclusively, while clinical tests for year and a half have proven its value in inflammatory conditions of the mouth.

R-10 is first of all a powerful germicide; it is an alterative; it is a sedative; it is a stimulant; it is an astringent; it is slightly anesthetic.

It has in its composition the following drugs:

Alcohol—Antiseptic.
Sulphur—Stimulant.
Hydrastis—Stimulant, astringent, antiseptic.
Thymol—Powerful germicide, true disinfectant.
Chloroform—Penetrating agent.
Iodine—Antiseptic, alterative.
Glycerine—Carrying agent, emollient.
Phenol—Disinfectant, antiseptic, analgesic.
Camphor—Circulatory stimulant.
Phenosulphonate Zinc—Astringent, antiseptic.

It will be seen that this formula is composed of tried and proven remedies, and their combination increases its value as a germicide to a marked degree. It does not stain the teeth or leave any bad after effects.

It is a proprietary medicine because its value depends on its being properly compounded—the very best drugs must be used; because it can be put up in bulk for much less than it could be compounded every time you need it; and because the druggist in order to stand back of and guarantee the preparation must feel that he can in some measure be remunerated for his work.

I apply this medicine to the gums and inject it into the pockets for a week or ten days, every other day. After the third or fourth day, if any inflammation exists, it indicates that your scaling has not been thorough at that point and your instrumentation should be repeated there.

Before applying the medicine, I wash the mouth out with a warm alkaline solution. A little warm salt water is all that is necessary. This is slightly antiseptic and is soothing and agreeable to the tissues.

### INTERNAL REMEDIES.

In cases where the symptoms point to Auto-intoxication, constipation, etc., I frequently prescribe some of the saline laxatives in small doses. They cleans the alimentary tract and eliminate irritating toxins. No doubt, occasions arise where the patient should be put under the care of a family physician. The dentist can do much good in this way by warning patients of diseases which he thinks may be present.

### INSTRUCTIONS.

Instruct the patient to use nothing during the treatment except a warm saline solution with which to clean the teeth. Also instruct the patient to rinse the mouth every night with milk of magnesia. This affords protection for the teeth at night when fermentation is most pronouneed. I regard this of great value, as I have seen wonderful results from its continued use.

While I do not object to the use of tooth powders, pastes, mouth washes, etc., I think they are of little value except as breath perfumes.

The tooth brush, during the period of treatment, should be softened with warm water before using. A good substitute for the bristle brush is a rubber finger brush; this, together with floss silk properly used, will clean the teeth. All patients should be instructed to carefully massage the gums towards the gingival margins.

### MECHANICAL TREATMENT.

Teeth that will not tighten after the deposits have been removed must be made firm in their sockets by attaching them in some manner to others that are firm. Whether these teeth can be made comfortable and useful depends upon the judgment of the operator. Of course, in some instances teeth are so loose, owing to absorption of alveolar process that no other course remains except resort to extraction. Many of these teeth can be used on permanent splints. Used in this manner they are just as useful and far more natural looking than artificial substitutes.

Where the lower incisors are loose a permanent splint can best be made by removing the pulps, fitting irridioplatinum posts to the canals, makin wax discs for two teeth at a time, casting the splint in sections, which are afterwards soldered together, and the piece cemented to place. I have several cases of this character where I extracted all four of the lower incisors and replaced them on the splint.

Bridge work can be made to do its part in replacing teeth that have been extracted. Special attention in the construction of this work should be given the self-cleansing spaces so the abutments can be kept clean. I believe that in most cases fixed bridgework will be of more value than any form of removable work so far devised.

To summarize, I know many cases of pyorrhea are curable—if incurable, great good and relief may be afforded the patient and the evil day of an edentulous mouth put off for a long time. If a case of pyorrhea can be cured at all, it can be cured in two or three weeks time; whether it remains cured, depends upon the care given by the patient. This care would include a visit to the dentist at least every six months for an examination.

I am firmly of the opinion that it is the duty of every dentist to know how to handle this disease, not alone for his patient's good but for the protection of his own work. Let me cite an instance in support of this: I had a patient who applied to me for some work. As is my custom, I insisted that the teeth must be properly cleaned before I would do the work. He consented. I did my best and thought his mouth was perfectly healthy. I made and inserted a small bridge—gold crown on molar, one bicuspid dummy, one inlay attachment in distal surface of first bicuspid. He wore it for a day or two and came back complaining that the bridge was too high. I ground it down, but the bridge still felt uncomfortable. This continued for about two weeks when the gum over the bicuspid began to swell a little. All I had to do was to look as wise as possible under the circumstances and pronounce it a pericimental abscess. I went after that tooth again with my files and polished the root surface thoroughly, as I thought, but I had to do this the second time before the abscess healed. Now the bridge is perfectly comfortable, and the gum looks as natural as if nothing had ever happened. If I had believed that pyorrhea was incúrable; that a pericemental abscess could not be relieved, my patient would have lost that bridge, and his confidence in me would have departed.

I hope that every one of us may see our duty and realize that the cleaning of teeth, the treating of pyorrhea, or whatever it may be termed is the foundation of good dentistry.

